# Risk of asthma exacerbation associated with nonsteroidal anti-inflammatory drugs in childhood asthma: A nationwide population-based cohort study in Taiwan

**DOI:** 10.1097/MD.0000000000005109

**Published:** 2016-10-14

**Authors:** Pei-Chia Lo, Yueh-Ting Tsai, Shun-Ku Lin, Jung-Nien Lai

**Affiliations:** a Institute of Traditional Medicine, School of Medicine, National Yang-Ming University; b Department of Chinese Medicine, Taipei City Hospital, Renai Branch, Taipei; c School of Chinese Medicine, College of Chinese Medicine, China Medical University; d Department of Chinese Medicine, China Medical University Hospital, Taichung, Taiwan.

**Keywords:** aspirin, asthma, National Health Insurance Research Database, NSAIDs, pediatrics

## Abstract

Patients allergic to aspirin or nonsteroidal anti-inflammatory drugs (NSAIDs) who develop respiratory reactions such as bronchospasm or asthma exacerbation have aspirin-induced asthma or NSAIDs-exacerbated respiratory disease. However, large-scale studies have not been conducted to investigate the risk of aspirin/NSAIDs exposure in children with asthma. Therefore, this study evaluated the relationship between aspirin/NSAIDs and the risk of asthma exacerbation in children with asthma.

This retrospective cohort study was conducted using the data of 1 million random beneficiaries of the Taiwan National Health Insurance program between 1997 and 2012. Children aged ≦18 years diagnosed with asthma by physicians were enrolled. The study population was divided into the index group (concurrently using antiasthmatic agents and NSAIDs patients) and reference group (using antiasthmatic drugs alone), and the relative risks (RRs) of hospitalizations resulting from asthma exacerbation in both groups were estimated.

The rate of asthma exacerbation was higher in the index group than the reference group, resulting in asthma-related hospitalizations (RR: 1.49, 95% confidence interval [CI]: 1.37–1.61; adjusted RR: 1.41, 95% CI: 1.30–1.53). Short-term aspirin, ibuprofen, and diclofenac use probably correlated with asthma exacerbation in children with asthma. No association between long-term aspirin, ibuprofen, and diclofenac consumption and the risk of asthma exacerbation was identified in this study.

## Abstract 2

Supplemental Digital Content is available in the text

## 1 Introduction

Aspirin-induced asthma (AIA) or nonsteroidal anti-inflammatory drug (NSAID)-exacerbated respiratory disease (NERD) is defined as hypersensitivity to aspirin/NSAIDs, causing respiratory-related symptoms such as bronchospasms, acute asthma exacerbation (lower airway), and severe asthma morbidity.^[1,2]^ AIA/NERD was traditionally diagnosed using the patient's history and an aspirin/NSAIDs oral provocation test.^[2]^ According to a meta-analysis, the prevalence of AIA among children with asthma is 5%^[3]^ and that of NERD among normal children is approximately 0.3%.^[4]^ However, limited data are available on the nationwide prevalence of NERD among children with asthma.

According to our previous study, approximately 60% of adult patients with asthma were concurrently prescribed antiasthmatic drugs and NSAIDs in Taiwan.^[5]^ Paradoxically, NSAIDs, including aspirin, possibly cause asthma exacerbations, particularly in patients allergic to these drugs.^[6]^ Aspirin/NSAIDs inhibit cyclooxygenase (COX) and reduce prostaglandin synthesis, thereby reducing fever and relieving pain and inflammation. However, inhibition of the COX pathway activates the lipoxygenase pathway, leading to increased leukotriene synthesis and risk of bronchospasms or asthma exacerbation, which was definitively first described by Andrzej Szczeklik.^[7,8]^

Currently, aspirin is not recommended for children <12 years of age because it causes severe side effects such as the Reye syndrome.^[9,10]^ Therefore, because of safety concerns for children, physicians prescribe NSAIDs instead of aspirin. However, studies have indicated that cross-sensitivity reactions occur with the use of both aspirin and NSAIDs, particularly ibuprofen, naproxen, and diclofenac.^[3,11]^ To date, NSAIDs are the most widely prescribed antipyretics in the world; however, few studies have focused on large-scale analysis of NSAID subtype exposure in children with asthma. Therefore, this study investigated the relationship between aspirin/NSAIDs and risk of asthma exacerbation in children. It is necessary to take precautions while treating children with asthma who might be sensitive to NSAIDs in order to reduce NSAIDs abuse.

## 2 Methods

### 2.1 Data resources

The present research was a retrospective cohort study. Of the 23 million patients enrolled in the Taiwan National Health Insurance (NHI) program, data of 1 million patients between January 1, 1997 and December 31, 2012 were obtained from the NHI Research Database (NHIRD). All the insurance information, such as date of birth, gender, age, region, salary, and medical records, including diagnosis, treatment, medication, and hospitalization date, are recorded in this database.^[12,13]^ To protect patient identity, patient identity numbers are encrypted by the NHIRD. The study protocol was reviewed and approved by the Ethics Review Board of Taipei City Hospital, Taiwan, in 2014.

### 2.2 Study population

**Figure 1:**
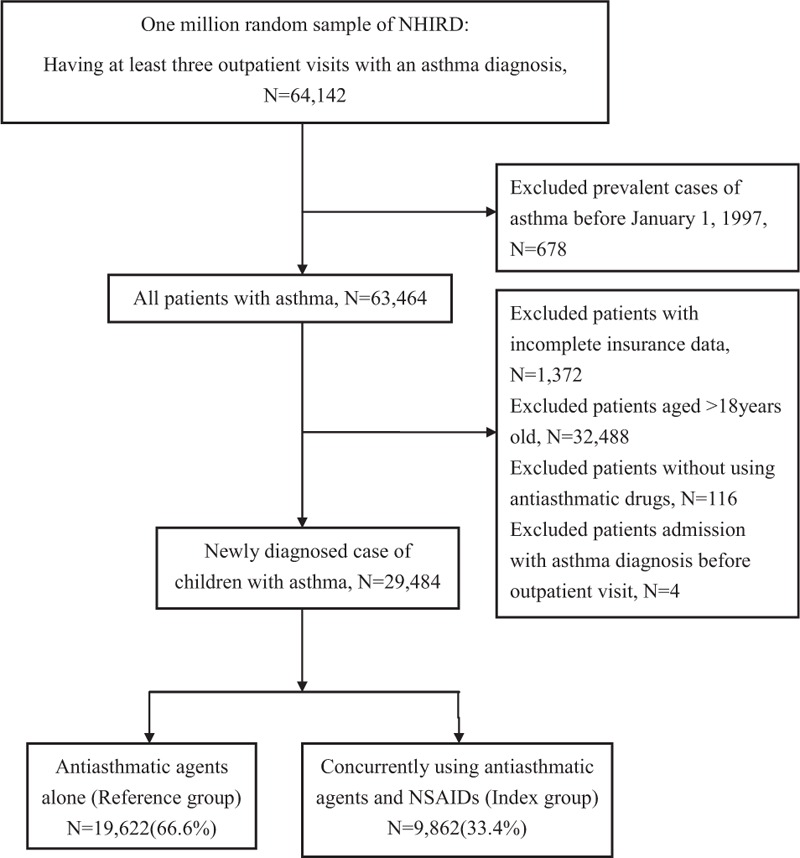
Recruitment flowchart of patients from the random sample of 1 million obtained from the National Health Insurance Research Database from 1997 to 2012 in Taiwan.

Figure 1 shows the recruitment procedure of the study population. We included patients who had been diagnosed with asthma more than 3 times during outpatient visits using the diagnosis code of asthma in the International Classification of Disease, Ninth Revision, Clinical Modification (ICD-9-CM) (493.0).^[14]^ We excluded patients with an asthma diagnosis before January 1, 1997 (n = 678), patients with incomplete insurance data (n = 1372), or who were >18 years (n = 32,488). In addition, to confirm that the study population only included children with asthma, patients who were not using antiasthmatic agents (n = 116) were excluded. Antiasthmatic agents, such as controllers (glucocorticosteroids, long-acting β2-agonists, mast cell stabilizers, antileukotriene, and anti-immunoglobulin E monoclonal antibody) and relievers (short-acting β2-agonists and short-acting anticholinergics), were prescribed on the basis of the treatment guidelines published by the Global Initiative for Asthma.^[15,16]^ Regarding asthma medication, we included only inhaled corticosteroids such as fluticasone, beclomethasone, and budesonide; nasal sprays, tablets, and steroid injections were excluded (Appendix 2).

Patients who had been previously admitted because of asthma before the outpatient visit and were diagnosed with asthma (n = 4) were excluded to ensure that the causal relationship can be analyzed in this study. We divided our study population into 2 groups: the reference group (using antiasthmatic agents alone) and the index group (concurrently using antiasthmatic agents and NSAIDs patients). In this study, “concurrently using antiasthmatic agents and NSAIDs” means that patients were prescribed antiasthmatic drugs and NSAIDs on the same day.

### 2.3 Asthma hospitalization

The start of the observation period was the first diagnosis date of asthma. The endpoint was either when patients were hospitalized with asthma or the last medical record before December 31, 2012. Only children hospitalized with first-time acute asthma exacerbation were enrolled. Respiratory infection was a major confounder in this study; thus, asthmatic children who were admitted with an acute respiratory illness associated with upper airway infections (ICD-9: 465), common cold (ICD-9: 460), and pneumonia or bronchopneumonia (ICD-9: 480–485) were excluded (Appendix 1), meaning that all patients with asthma-related hospitalization in the present study were admitted solely because of asthma exacerbations.

### 2.4 Study variables

Based on a previous study,^[5]^ a series of demographic factors were selected as variables, including gender, age, insured region, classification of asthma, and comorbidity. According to the clinical features, asthma was classified into the following 4 categories: extrinsic asthma ICD-9: 493.0 (atopic asthma), intrinsic asthma ICD-9: 493.1 (nonatopic asthma), chronic obstructive asthma ICD-9: 493.2, and unspecified asthma ICD-9: 493.9.^[14]^ Allergic rhinitis, atopic dermatitis, and urticaria are some of the comorbidities that are commonly clinically associated with asthma.^[17–19]^ In addition, gastroesophageal reflux disease,^[20]^ which has been correlated with asthma, was included as a variable (Appendix 1). Exposure to exhaust fumes in industrial areas or from traffic emissions can increase the risk of asthma exacerbation and hospitalization,^[21,22]^ and the exposure volume is correlated highly with the living area.^[23,24]^ A previous study reported that air quality differs by region in Taiwan, with eastern Taiwan having higher air quality than do other regions.^[23]^ In this study, we consider the insured region as a demographic variable representing the living environment because this variable is an appropriate representative of the long-term air pollution exposure volume. The children were classified into 1 of 7 regions on the basis of their insured region: Taipei City, Kaohsiung City, northern Taiwan, central Taiwan, eastern Taiwan, southern Taiwan, and outlying islands. Each of these regions has different levels of air pollution.

### 2.5 Statistical analysis

Differences in the demographic characteristics in the index and reference groups were analyzed using chi-squared test (Table 1). Relative risk (RR) was used as a measure of the relationship between an insured region and the risk of asthma-related hospitalization (Table 2). The risks of NSAIDs exposure and asthma-related hospitalizations were estimated as RRs in the 2 groups, and a log-binomial regression model was used to estimate the adjusted RR (aRR; Table 3). In addition, the model was stratified by the duration and cost (i.e., sum of all medical expenses incurred during the hospitalization, inclusive of examination fee, treatment fee, drug fee, special medical fee, special medical supply fee, and ward fee^[25]^) of asthma-related hospitalization.

**Table 1:**
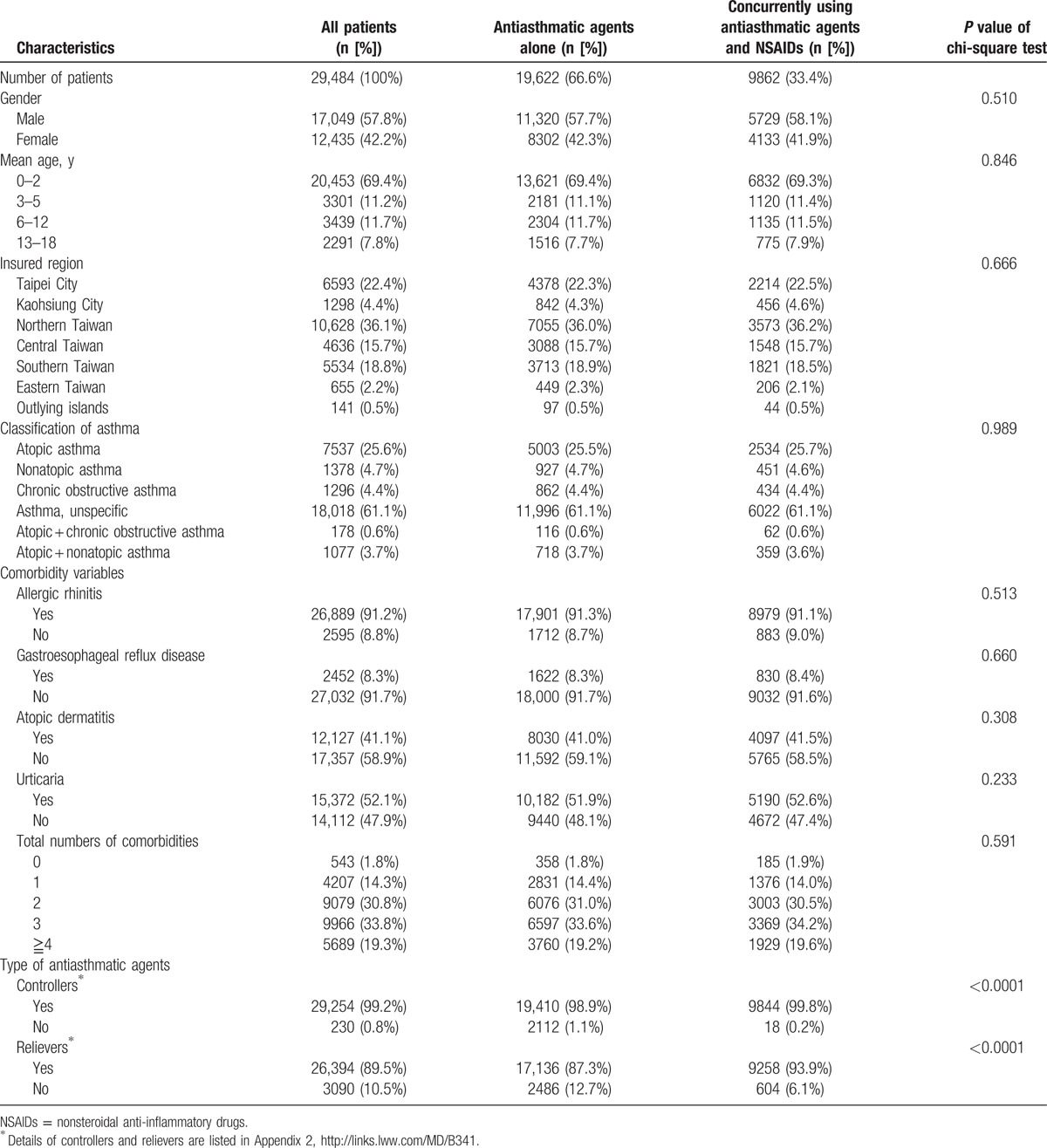
Demographic characteristics of the children with asthma from 1997 to 2012 in Taiwan.

**Table 2:**
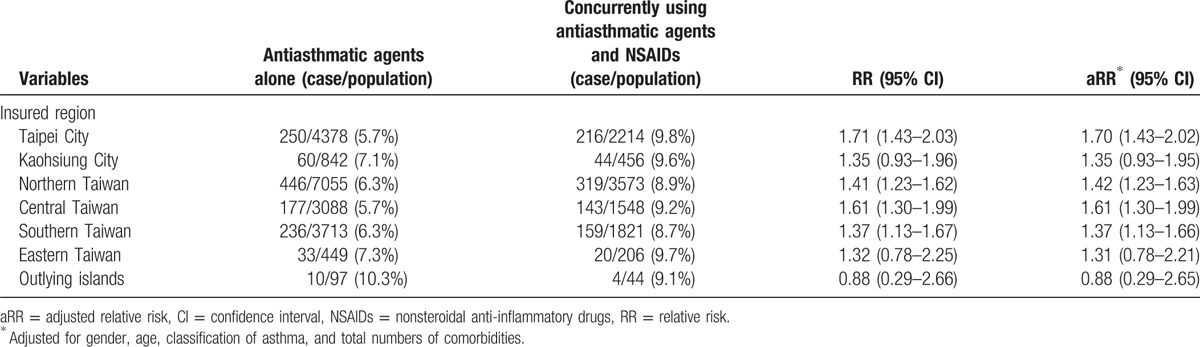
Estimated relative risk of asthma exacerbation for children in different insured regions in Taiwan from 1997 to 2012.

**Table 3:**
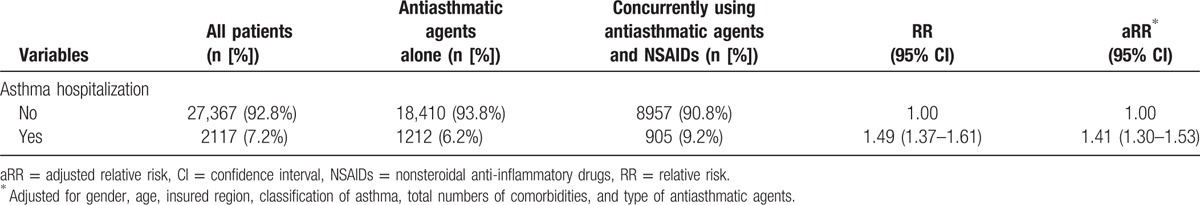
Asthma exacerbation resulting in asthma-related hospitalization in children with asthma in Taiwan from 1997 to 2012.

To examine the short-term risk of different NSAIDs, the exposure time was stratified into 3 periods: 0, 1 to 2, and ≧3 days before asthma-related hospitalization. All NSAID prescription records were obtained from outpatient visit records, and the logistic regression model was used to calculate the adjusted odds ratio (aOR). Furthermore, to examine the dose–response effect of the NSAIDs, cumulative days of NSAIDs exposure were used, and the exposure time was stratified into different periods: 0, 1 to 7, 8 to 30, 31 to 90, and >90 days. All statistical analyses were performed using SAS version 9.4 software (SAS Institute Inc., Cary, NC). *P* < 0.05 was considered statistically significant and was calculated against 95% confidence intervals (CIs).

## 3 Results

Of the total 29,484 patients, 19,622 (66.6%) patients used antiasthmatic agents alone (reference group), and 9862 (33.4%) patients concurrently used antiasthmatic agents with NSAIDs (index group). By the end of the study period, 1212 patients (6.2%) in the reference group were eventually hospitalized because of asthma, whereas 905 patients (9.2%) in the index group were hospitalized because of asthma (Table 3).

Approximately 80% of children with asthma develop symptoms before 5 years of age.^[26,27]^ In our study, approximately 80.6% of children had been diagnosed with asthma before the age of 5 years (Table 1), indicating that asthma affects children in their early development stage. Consistent with previous studies,^[28,29]^ the prevalence of asthma was higher in boys (57.8%) than in girls (42.2%). The highest density of children with asthma was found in Taipei and northern Taiwan, accounting for 58.5% of the population. Furthermore, atopic asthma accounts for approximately a quarter of patients with asthma (25.6%); however, nonatopic asthma was only present in 4.7% of patients with asthma. Allergic rhinitis, observed in up to 91.2% of our study population, was highly associated with asthma. Other comorbidities, including atopic dermatitis and urticaria, were present in 41% and 52% of children with asthma. There was no significant difference in the demographic characteristics between the 2 groups.

In most regions, the index group had a higher RR of asthma-related hospitalization than did the reference group (Table 2). The RR of asthma-related hospitalization with asthma exacerbation in the 2 groups is shown in Table 3. The index group had a higher RR of asthma-related hospitalization (RR: 1.49, 95% CI: 1.37–1.61; aRR: 1.41, 95% CI: 1.30–1.53) than did the reference group. Despite stratifying the duration of hospital stays and cost of hospitalization, the index group showed a higher RR of asthma exacerbation than did the reference group (Table 4).

**Table 4:**
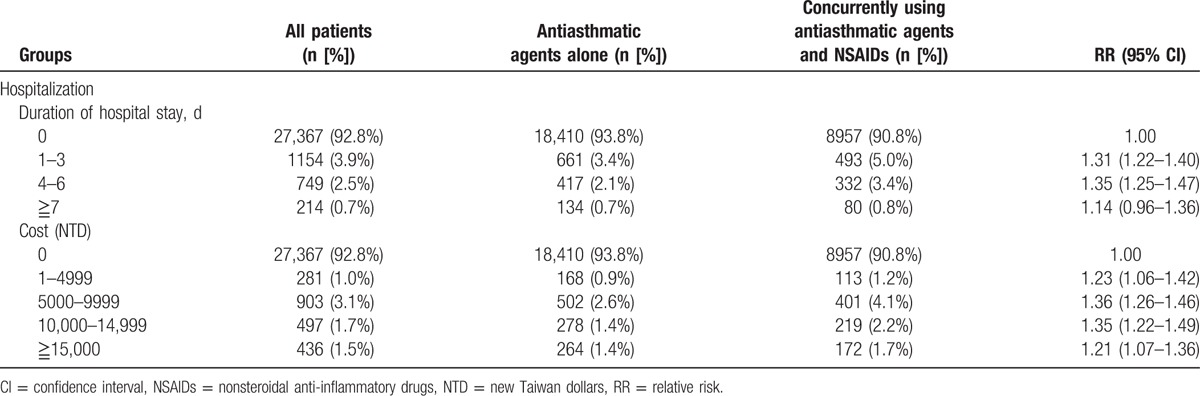
Stratified hospital days and cost of asthma-related hospitalization in children with asthma from 1997 to 2012 in Taiwan.

Details of the short-term use of coprescribed NSAIDs in prehospitalized children are listed in Table 5. After adjustment for sex, age, insured region, asthma classification, number of comorbidities, and type of antiasthmatic agent, ibuprofen had the highest usage rate in 1 to 2 days before asthma-related hospital admission. Compared with the reference group, the index group had a higher percentage of exposure to ibuprofen, diclofenac, in 1 to 2 days before asthma-related hospital admission (ibuprofen: aOR: 3.65, 95% CI: 1.98–6.74; diclofenac: aOR: 2.90, 95% CI: 1.23–6.84).

**Table 5:**
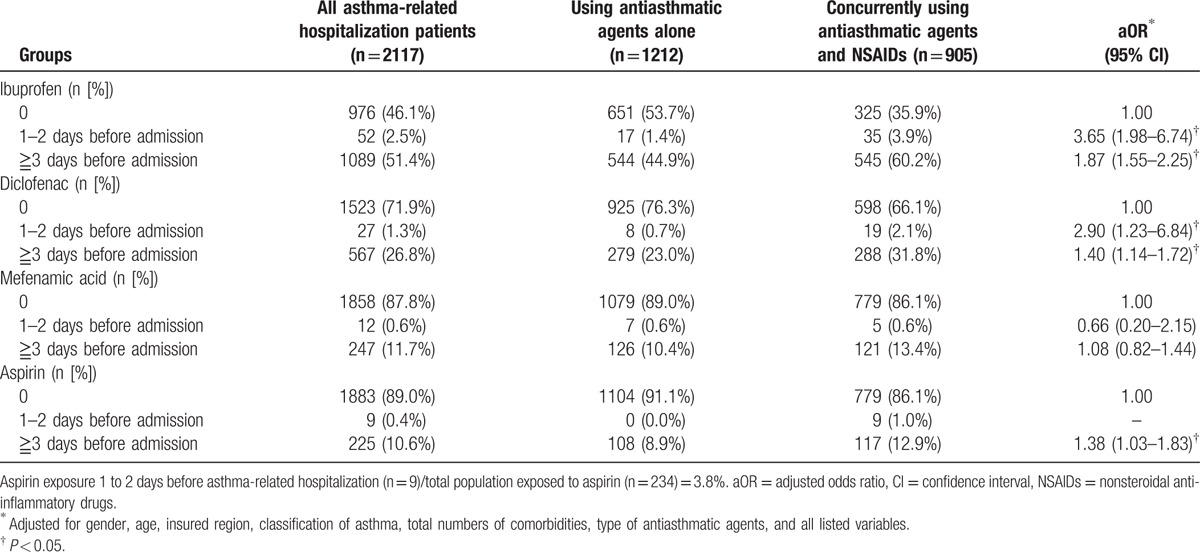
NSAIDs exposure in children with asthma before asthma hospitalization.

The long-term exposure of NSAID subtype that affects children with asthma is outlined in Table 6. Compared with other NSAIDs, patients with cumulative exposure to diclofenac, aspirin, and ketoprofen were at a nonsignificantly higher risk of asthma-related hospitalization.

**Table 6:**
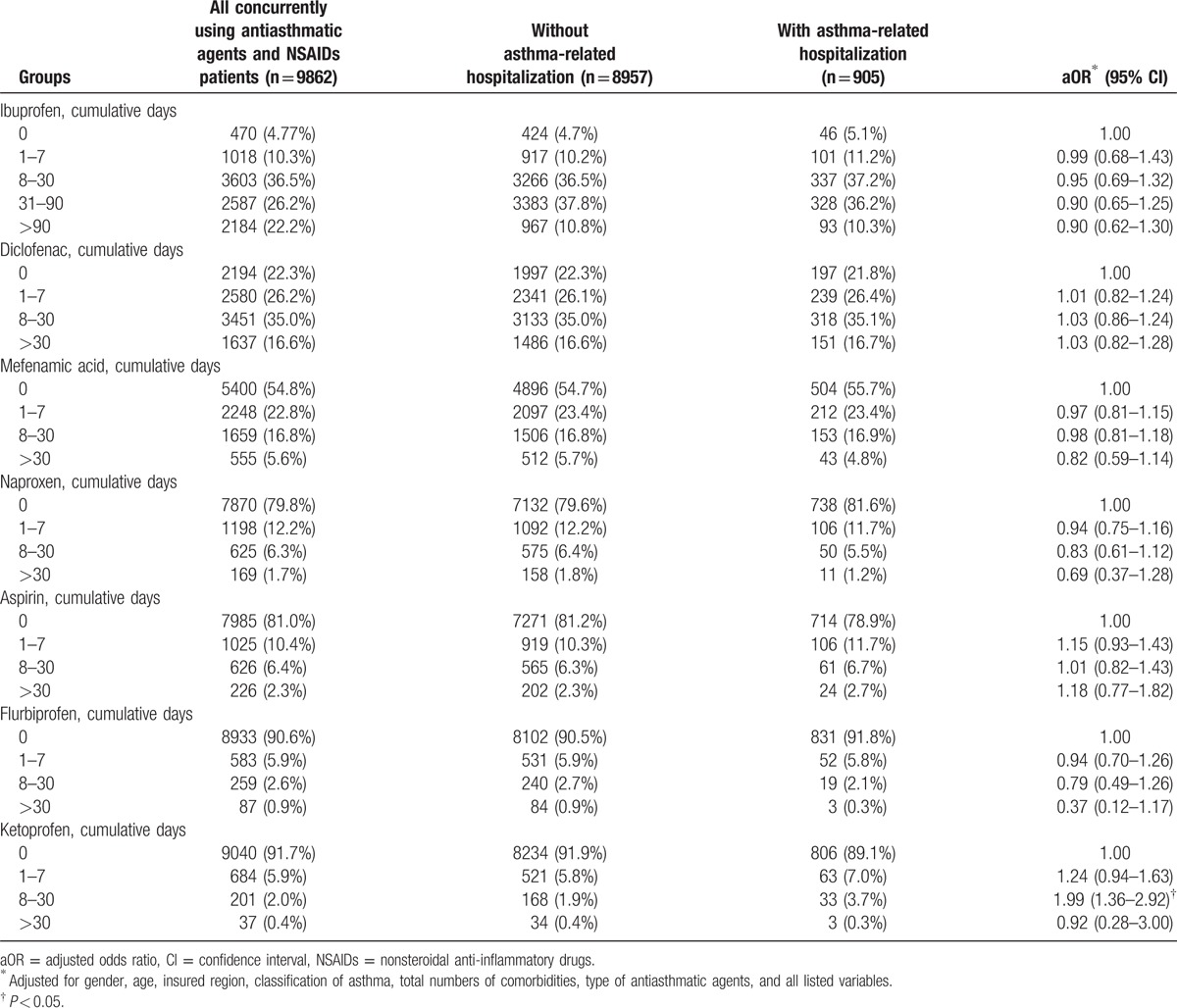
Cumulative days of NSAIDs exposure in the index group (concurrently using antiasthmatic agents and NSAIDs patients).

## 4 Discussion

According to our literature review, this is the first study to use a nationwide population-based cohort to document the coadministration of antiasthmatic agents and aspirin/NSAIDs in children with asthma in Taiwan. Children with asthma who were receiving antiasthmatic drug prescriptions frequently consumed NSAIDs. From 1997 to 2012, >33% of children whose asthma was under control were being prescribed an antiasthmatic drug and a NSAID on the same day for relieving pain or fever-like symptoms. Numerous problems are associated with asthma when conventional antiasthma medicines—which decrease resistance in the respiratory airway and increase airflow to the lungs—and NSAIDs—which may cause bronchospasms—are taken simultaneously by children with asthma, resulting in an unpredictable outcome for asthma control.

This study demonstrates that NSAIDs use was associated with an increased risk of asthma exacerbation. In general, NSAID-induced bronchospasm develops within 30 to 180 minutes (sometimes up to 24 hours) after drug ingestion,^[30]^ possibly precipitating the asthma exacerbation. To examine the short-term effects of NSAIDs on asthma exacerbation, we analyzed the population hospitalized for asthma and defined the NSAID exposure time 1 to 2 days before hospitalization as our assessment point. During the study period, 234 children with asthma were exposed to aspirin, and 9 of them developed an acute asthma exacerbation within 48 hours after aspirin use. Therefore, it can be inferred that the incidence of AIA among children with asthma in Taiwan was approximately 3.8%, which, however, in a previous study was 5%.^[3]^ This difference can be attributed to the varying definitions of asthma exacerbation. It is not surprising that the prevalence rate of AIA based on aspirin provocation tests is higher than that based on the requirement for hospital admission. Both physicians and parents should be aware of the association between the concurrent use of antiasthmatic agents and aspirin and potential risk of acute asthma exacerbation in children with asthma. Therefore, early diagnosis is essential in sensitive populations, and further management is required to prevent severe AIA exacerbation among vulnerable patients with asthma in Taiwan.

In addition, the study results demonstrate that ibuprofen, diclofenac, mefenamic acid, naproxen, ketoprofen, and flurbiprofen were the 6 most prescribed NSAIDs for relieving pain or fever-like symptoms among children with asthma in Taiwan, and short-term use of ibuprofen and diclofenac increases the risk of asthma-related hospitalization. Because ibuprofen has stronger analgesic effects than does acetaminophen,^[31]^ it has been the most frequently prescribed for treating fever or relieving pain in children. However, the prevalence of ibuprofen-sensitive asthma is 2% among 6- to 18-year-old children.^[32]^ Furthermore, using the calculation method of cumulative exposure time, the European Safety Of non-Steroidal anti-inflammatory drugs project) indicated that ibuprofen was associated with the adverse effect of asthma exacerbation (2-fold).^[33]^ A previous in vitro study indicated that NSAIDs weaken the immune system by decreasing antibody synthesis, resulting in lowering host defense.^[34]^ However, the accumulation calculation method of our study did not observe the relationship between chronic exposure to ibuprofen and long-term adverse effects in asthmatic children. Furthermore, an oral provocation test indicated that ingestion of diclofenac can cause a 15% reduction in lung function in children with asthma aged 6 to 15 years.^[35]^ Another report studying the cross-sensitivity in NSAIDs indicated that diclofenac reduced 16% to 25% of peak expiratory flow.^[3]^ In our study, the short-term use of diclofenac increased the risk of asthma exacerbation in children. Diclofenac is one of the top NSAIDs that causes adverse effects in Taiwan, accounting for 3.15% of all the drug adverse events in 2012.^[36]^

To our knowledge, this is one of the first observational studies to prove that the risk of asthma exacerbation is associated with the short use of NSAIDs in children whose asthma is under control. This study urges the physicians to reassess their treatment strategies for fever in children with asthma. In addition, further research on optimal treatments and the long-term outcomes of NSAID-induced asthma exacerbation are warranted.

The present study has 4 limitations. First, this study did not include some over-the-counter NSAIDs available in Taiwan, meaning that the frequency of NSAIDs use might have been underestimated. However, because the NHI system covers all prescriptions by qualified physicians after careful examinations, providing affordable, accessible, and convenient asthma healthcare, the likelihood of parents purchasing over-the-counter NSAIDs is not high. Second, because the medical records were retrospective, we were unable to ensure whether children with asthma had taken their prescribed NSAIDs. However, all NSAIDs were recommended based on expert opinions; therefore, the compliance of NSAIDs in children with asthma is assumed to be high. Third, owing to the lack of actual clinical data, we are unable to draw any conclusions on how the severity of asthma-related symptoms is associated with NSAIDs use. Finally, the lack of personal air pollution exposure data precluded an analysis of the association between individual exposure and asthma-related hospitalization. Therefore, the aRR of asthma exacerbation for each insured region was used to determine the effects of air pollution on the asthmatic children. Traffic is the major source of air pollutants in Taipei,^[37]^ whereas in central Taiwan, fine particles are produced primarily by thermal power plants^[23]^; a higher RR was observed in the effect estimates for northern Taiwan than for central Taiwan, indicating that the type and amount of air pollutants differentially influence the risk of asthma exacerbation in children taking antiasthma medication alone and in those receiving a combination of antiasthma medication and NSAIDs in Taiwan. Therefore, we adjusted for the insured regions in the final analysis. Secondhand smoking can also be a risk factor for asthma exacerbation^[38]^; the Taiwan government prohibited smoking in public places, including schools, nurseries, restaurants, and kindergartens, since 1997.^[39]^ However, the NHIRD does not record data on indoor air pollution; hence, we could not adjust for exposure to secondhand smoke; this means that the exposure assessment in this study is likely to have underestimated the magnitude of the investigated association but is not likely to have introduced a positive bias.

In conclusion, our results reveal that in Taiwan, NSAIDs and antiasthmatic agents are generally coprescribed, and a certain proportion of children with asthma are allergic to aspirin/NSAIDs. Short-term aspirin, ibuprofen, and diclofenac consumption is probably correlated with asthma exacerbation. Long-term aspirin, ibuprofen, and diclofenac consumption were not related to asthma-related hospitalization.

## Supplementary Material

Supplemental Digital Content
