# Supplementary material for: Risk of asthma exacerbation associated with nonsteroidal anti-inflammatory drugs in childhood asthma: A nationwide population-based cohort study in Taiwan
Source: Medicine (Baltimore). 2016 Oct 14;95(41):e5109. doi: 10.1097/MD.0000000000005109 (PMC5072955; doi:10.1097/MD.0000000000005109)
Supplement: Supplemental Digital Content [file medi-95-e5109-s001.doc]

**Appendix 1**. International Classification of Diseases, Ninth Revision (ICD–9), codes of comorbidity diseases used in this study.

| **Diseases** | **ICD–9 Codes** |
| --- | --- |
| Asthma  Extrinsic asthma (Atopic asthma)  Intrinsic asthma (Non atopic asthma)  Chronic obstructive asthma  (with obstructive pulmonary disease)  Asthma, unspecified | 493  493.00–493.02  493.10–493.12  493.20–493.22  493.90–493.92 |
| **Comorbidity Diseases** | **ICD–9 Codes** |
| Acute nasopharyngitis[common cold]  Acute respiratory infection  Allergic rhinitis  Viral pneumonia  Pneumococcal pneumonia  Other bacterial pneumonia  Pneumonia due to other specified organism  Pneumonia in infectious diseases classified elsewhere  Bronchopneumonia, organism unspecified  Gastro-esophageal reflux disease  Atopic dermatitis  Urticaria | 460  465  477  480  481  482  483  484  485  530.8  691  708 |

| **Appendix 2**. Details of anti-asthmatic agents and NSAIDs drugs used in this study.   | **Drug Classification** | **Detail** | | | --- | --- | --- | | **Anti-Asthmatic Drugs**  Reliever  Controller | Short-acting β2 agonists (SABA): salbutamol, terbutaline,  Short-acting anticholinergics: ipratropium bromide  Inhaled corticosteroids: fluticasone, beclomethasone, budesonide  Long-acting β2 agonists (LABA): salmeterol, fenoterol, metaproterenol,  Mast-cell stabilizers: cromolyn sodium  Antileukotriene: montelukast, zafirlukast  Anti-IgE monoclonal antibody: omalizumab | | | **NSAIDs** |  |  | | Salicylate (acetylated) | aspirin | | | Salicylate (non-acetylated) | diflunisal, salsalate | | | Propionic acids | naproxen, ibuprofen, ketoprofen, flurbiprofen, | | | Acetic acids | diclofenac, etodolac, ketorolac, indomethacin, tolmetin, sulindac | | | Oxicams | meloxicam, piroxicam | | | Fenamates | mefenamic acids | | | Nonacidic | nabumetone | | |  |
| --- | --- | --- | --- | --- | --- | --- | --- | --- | --- | --- | --- | --- | --- | --- | --- | --- | --- | --- | --- | --- | --- | --- | --- | --- | --- | --- | --- | --- | --- | --- | --- |
